# Role of Disease-Specific Incidence and Survival in Disparities in Life Expectancy in the United States

**DOI:** 10.1093/geroni/igab046.571

**Published:** 2021-12-17

**Authors:** Julia Kravchenko, Bin Yu, Igor Akushevich

**Affiliations:** 1 Duke University, Durham, North Carolina, United States; 2 Duke University, Duke University/Durham, North Carolina, United States

## Abstract

There are persisting geographic and racial disparities in life expectancy (LE) across the United States (US). We used 5% Medicare Claims data (2000-2017) to investigate how disease incidence and survival contribute to such disparities. Disease-specific hazard ratios (HRs) were calculated for Medicare beneficiaries living in the US states with the lowest LE (the states with the highest LE were used as a reference group), in gender- and race-/ethnicity-specific populations. Analysis of incidence showed that the greatest contribution to between-the-state disparities in LE was due to higher incidence (HRs≥1.30) of atherosclerosis, heart failure, influenza/pneumonia, Alzheimer’s disease, and lung cancer among older adults living in the states with the lowest LE. The list of diseases that contributed most to LE through the differences in their survival substantially differed from the above listed diseases: namely, diabetes, chronic ischemic heart disease, and cerebrovascular disease had HRs≥1.28 for their respective survival rates, with the highest HRs for lung cancer (HR=1.37, in females) and prostate cancer (HR=1.30). Respective race-/ethnicity-specific patterns of incidence and survival HRs were investigated and diseases contributed most to racial disparities in LE were identified. Study showed that when planning the strategies targeting between-the-state differences in LE in the US, it is important to address both 1) primary and secondary prevention for diseases demonstrating substantial differences in contributions of incidence, and 2) treatment choice, adherence to treatment, and comorbidities for diseases contributing to LE disparities predominantly through the differences in survival. Such strategies can be disease-, race-/ethnicity-, and geographic area-specific.

